# Supplementary figures and images for: Fluence-dependent degradation of fibrillar type I collagen by 222 nm far-UVC radiation
Source: PLoS One. 2024 Jan 2;19(1):e0292298. doi: 10.1371/journal.pone.0292298 (PMC10760738; doi:10.1371/journal.pone.0292298)

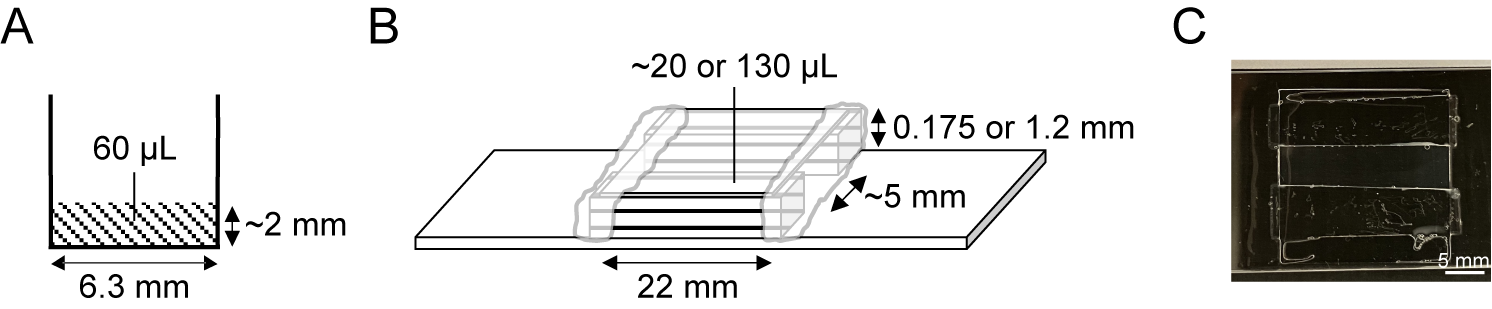

Supplement: S1 Fig — A: Single well of a 96-well plate. B: Microscopy sample chamber. C: Digital photograph of a representative microscopy sample chamber. (TIF) [file pone.0292298.s001.tif]

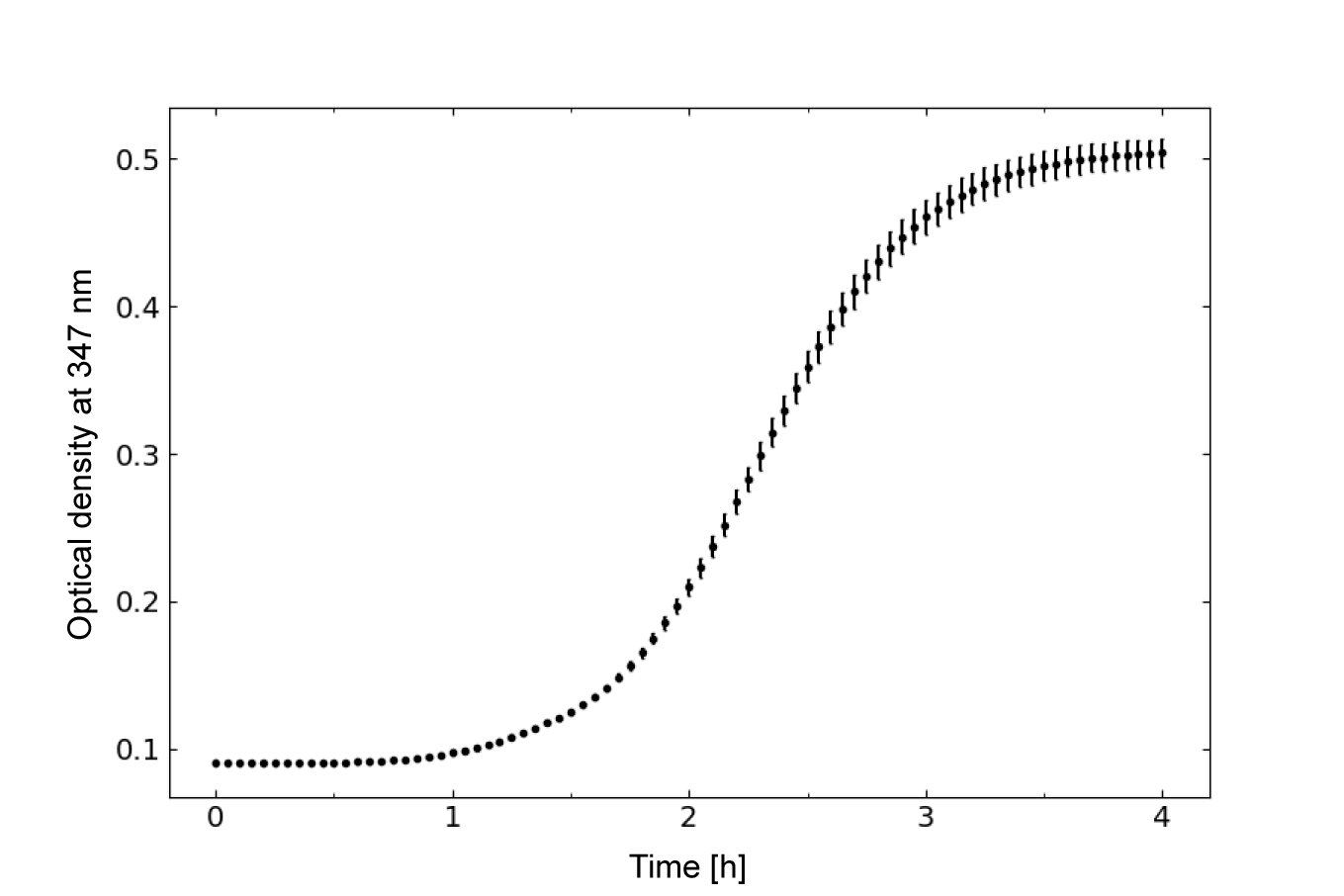

Supplement: S2 Fig — Error bars represent the standard error of the mean from three replicate measurements. (TIF) [file pone.0292298.s002.tif]

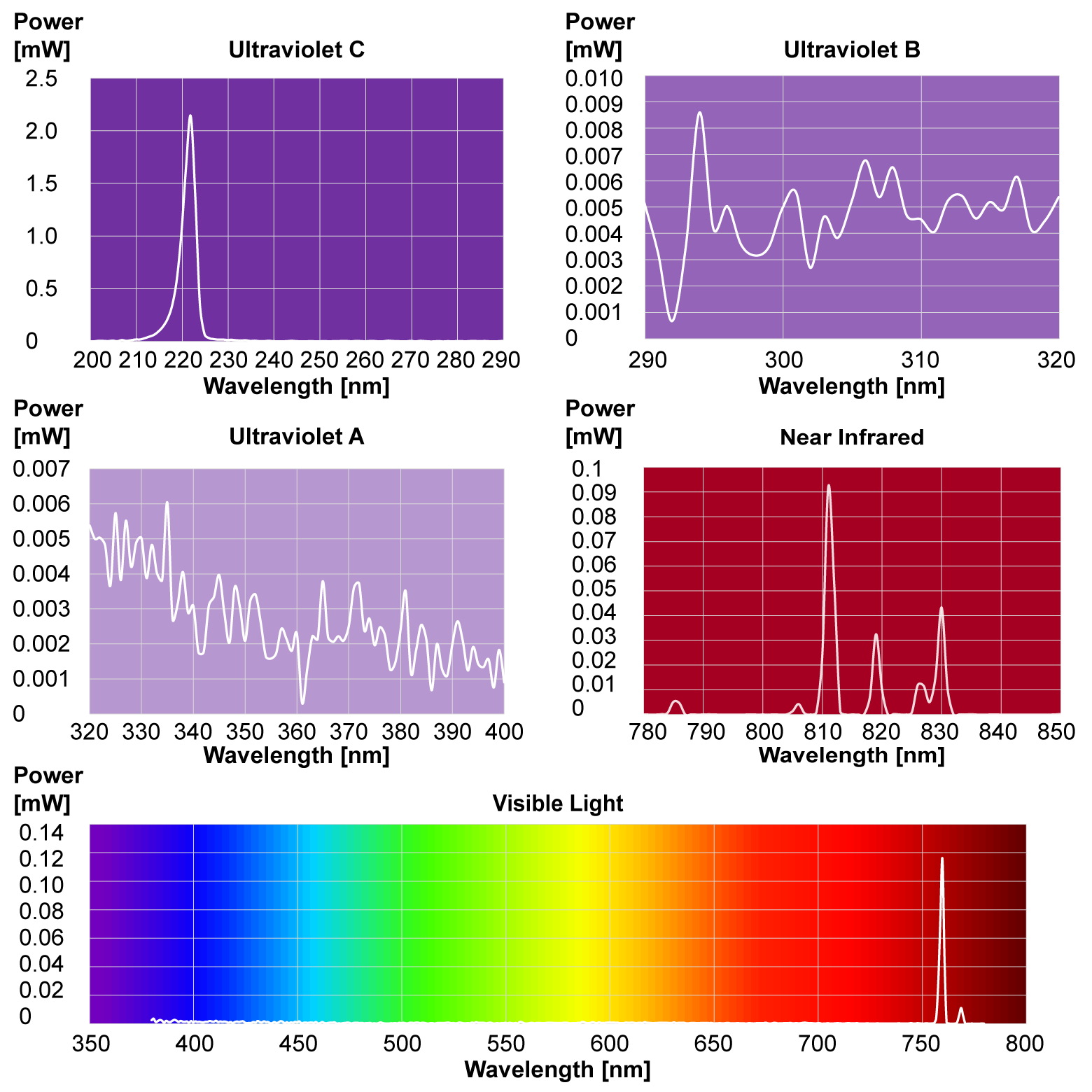

Supplement: S3 Fig — Data courtesy of Viso Systems (Copenhagen, Denmark). Figure adapted from Viso Systems. (TIF) [file pone.0292298.s003.tif]

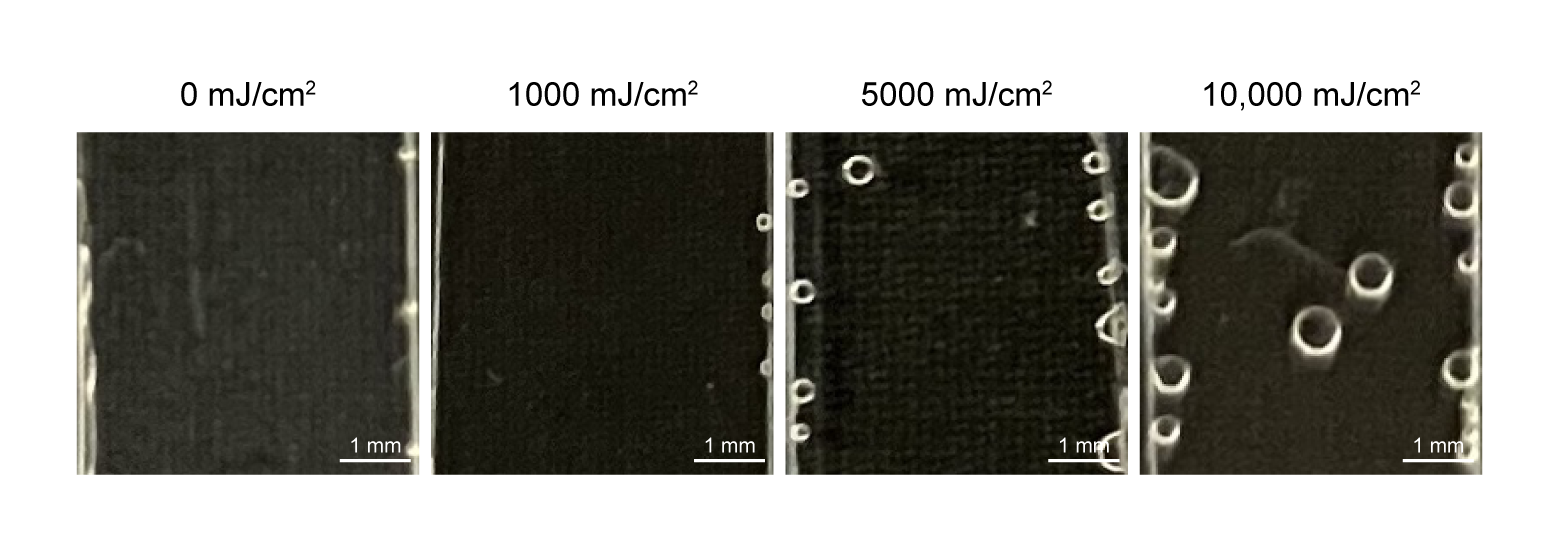

Supplement: S4 Fig — Gas bubbles are increasingly prevalent at higher fluences. (TIF) [file pone.0292298.s004.tif]

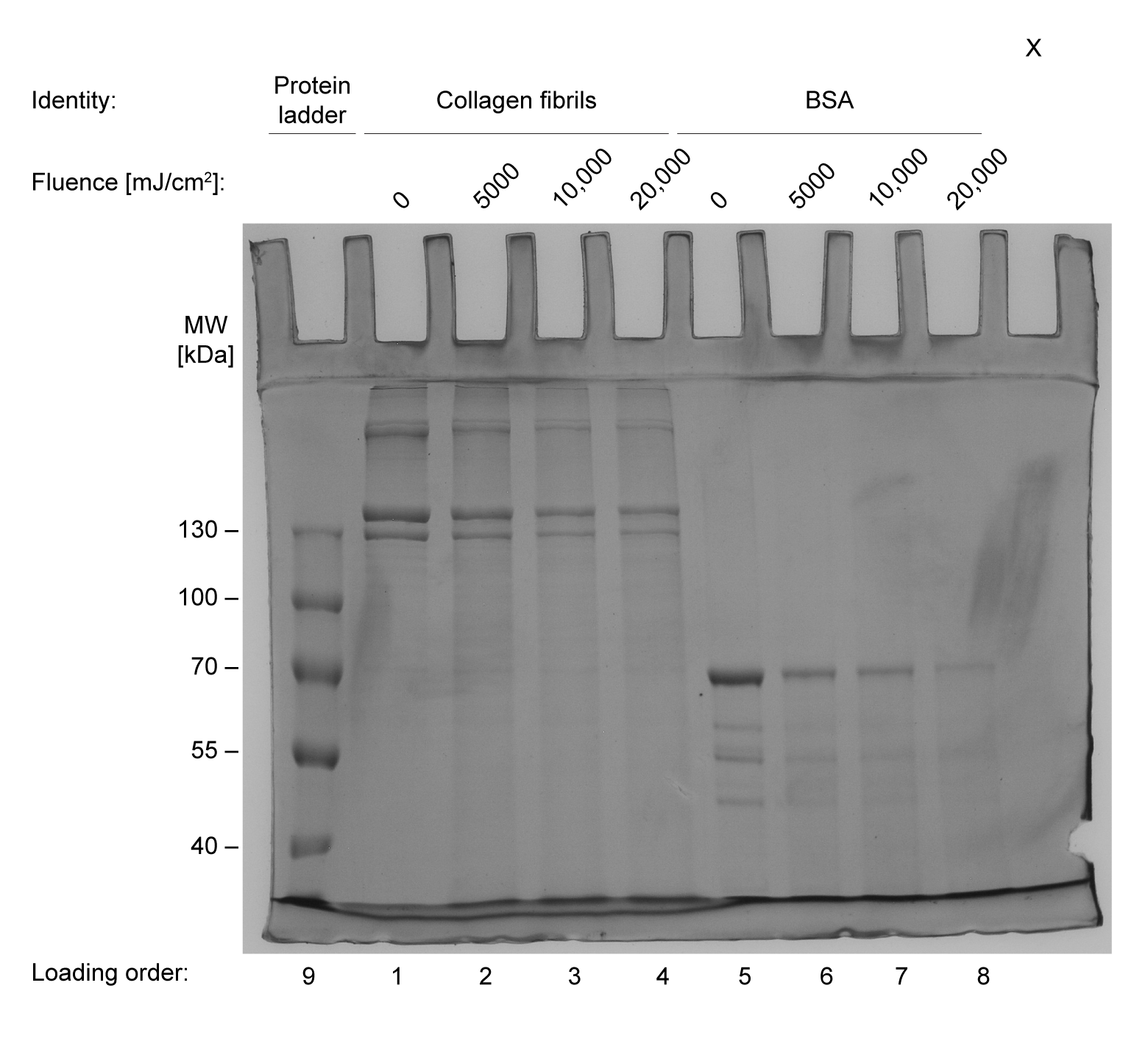

Supplement: S5 Fig — (TIF) [file pone.0292298.s005.tif]

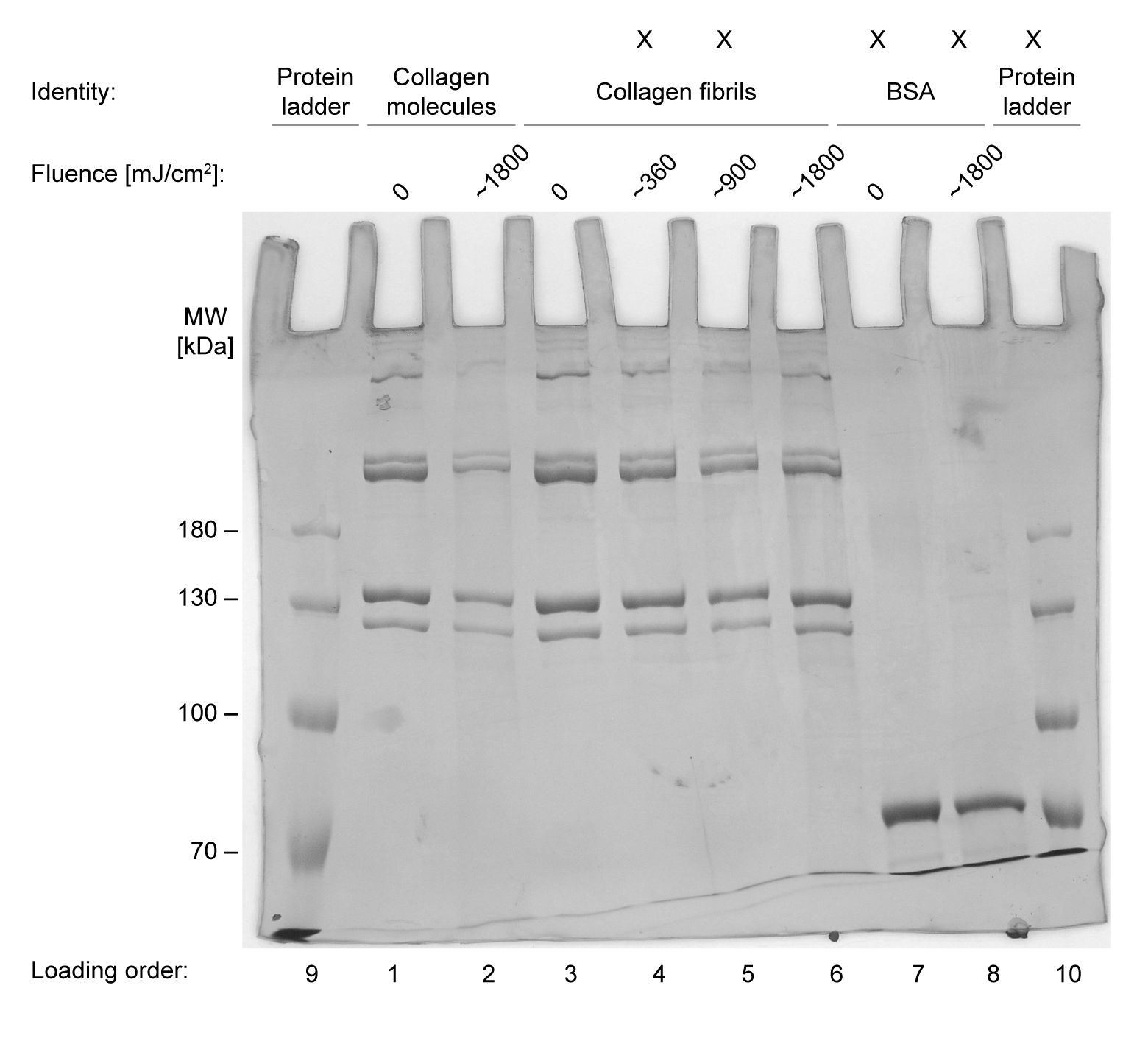

Supplement: S6 Fig — (TIF) [file pone.0292298.s006.tif]
